# Inducing cell death in vitro in cancer cells by targeted delivery of cytochrome c via a transferrin conjugate

**DOI:** 10.1371/journal.pone.0195542

**Published:** 2018-04-12

**Authors:** Manoj Saxena, Yamixa Delgado, Rohit Kumar Sharma, Shweta Sharma, Solimar Liz Ponce De León Guzmán, Arthur D. Tinoco, Kai Griebenow

**Affiliations:** 1 Department of Environmental Sciences, University of Puerto Rico, Rio Piedras Campus, San Juan, Puerto Rico, United States of America; 2 Department of Chemistry, University of Puerto Rico, Rio Piedras Campus, San Juan, Puerto Rico, United States of America; University of South Alabama Mitchell Cancer Institute, UNITED STATES

## Abstract

One of the major drawbacks of many of the currently used cancer drugs are off-target effects. Targeted delivery is one method to minimize such unwanted and detrimental events. To actively target lung cancer cells, we have developed a conjugate of the apoptosis inducing protein cytochrome c with transferrin because the transferrin receptor is overexpressed by many rapidly dividing cancer cells. Cytochrome c and transferrin were cross-linked with a redox sensitive disulfide bond for the intra-cellular release of the protein upon endocytosis by the transferrin receptor. Confocal results demonstrated the cellular uptake of the cytochrome c-transferrin conjugate by transferrin receptor overexpressing A549 lung cancer cells. Localization studies further validated that this conjugate escaped the endosome. Additionally, an in vitro assay showed that the conjugate could induce apoptosis by activating caspase-3. The neo-conjugate not only maintained an IC_50_ value similar to the well known drug cisplatin (50 μM) in A549 cancer cells but also was nontoxic to the normal lung (MRC5) cells. Our neo-conjugate holds promise for future development to target cancers with enhanced transferrin receptor expression.

## Introduction

Lung cancer is one of the leading causes of death in the USA with a 5 year survival rate of only about 18% [1]. Non-small cell lung carcinoma (NSCLC) accounts for ca. 85% of the lung cancer cases [2]. Platinum based drugs, including, cisplatin and carboplatin, are often used as the drug of choice in the treatment of lung cancer [3]. However, low solubility [4], the expulsion of drugs by Multi Drug Resistance (MDR) proteins [5], and development of resistance often limit their effectiveness [6]. The non-specificity of these drugs is a major challenge because these drugs not only kill cancer cells but are highly toxic to the normal cells too. For example, patients treated with cisplatin often suffer severe nephro-toxicity[2, 6]. Similarly another widely used small molecule anticancer drug, doxorubicin, is linked to cardiotoxicity in up to 30% of the patients [7]. Due to these detrimental off target effects, many conventional chemotherapeutic drugs have a low therapeutic index, which limits the effective dose that can be used without severely impacting the patient’s health.

The lack of specificity and flexibility to withstand modifications without loss of activity typical for small molecule drugs has lead to an increased interest in the development of cytotoxic protein drugs because they potentially could be readily modified given their large size and multiple potential attachment points [8, 9]. Cytochrome c (Cyt c) is one such protein that has attracted the attention of research groups due to its potential to be developed into a potent and selective anticancer drug [8, 10–12]. Cyt c is an evolutionarily conserved 12 kDa protein. It is long known to be part of the mitochondrial electron transport chain where it transfers an electron from complex III to complex IV. Its role as a trigger in apoptosis has been realized more recently [13]. Cyt c is released from mitochondria in response to external or internal events (e.g., DNA damage) and upon release into the cytoplasm can induce apoptosis [14]. Here Cyt c interacts with Apaf-1 to activate caspase-9 which in turn activates caspase-3 subsequently causing cell death [15]. However, in many cancers, proteins that regulate the outer mitochondrial membrane permeability for Cyt c release are rendered dysfunctional by mutations [16, 17]. Introducing Cyt c from an external source into the cytoplasm of cancer cells could help overcome the upstream blockage caused by mutations in the apoptotic pathways. This property of Cyt c to induce apoptosis has been explored in developing it as an anticancer drug [18]. Additionally, the use of Cyt c is attractive because the activation step of caspase-9 by Cyt c lies downstream in the apoptotic pathway making it less prone to inactivation by mutations [18].

However, like most other proteins, Cyt c cannot enter the cell making its delivery to the cytoplasm quite challenging [19]. To address this delivery issue a number of delivery systems, including lipid based systems and mesoporous silica, have been explored to deliver proteins into the cells [8, 19]. However, these systems still face many challenges like poor biocompatibility, immunogenicity, inactivation of the protein, and clearance by the immune system. An additional challenge to develop cytotoxic proteins like Cyt c as a cancer drug is their lack of specificity. In this case, Cyt c would induce apoptosis in any cell it is delivered to because the apoptosis machinery is ubiquitous. To overcome this challenge many receptor ligands have been explored to selectively target the drugs to cancer cells [20]. The transferrin receptor (TfR) is one such promising receptor candidate that binds iron(III)-bound serum transferrin (Tf) to supply iron from the blood into cells [21]. Due to their rapid growth cancer cells often have a much higher expression of Tfr to upkeep their increased demand for iron [22]. During the iron uptake cycle, diferric Tf binds to the cell surface TfR and the resulting Tf-receptor complex is endocytosed. Owing to the receptor’s higher expression in cancer cells, Tf has been conjugated to DNA, small molecules, and toxic proteins as a delivery system [22–25].

We decided to explore the potential of a Cyt c-Tf conjugate to target cancer cells for the induction of apoptosis. Because the cytosol is reducing in nature, a redox responsive crosslinker (Sulfo-LC-SPDP) was used to conjugate both proteins for smart release of the Cyt c into the cytoplasm [26]. The uptake of Cyt c-Tf conjugate was studied in A549 lung cancer cells expressing TfR by fluorescence microscopy. The in vitro cell viability assay shows that the Cyt c-Tf conjugate can induce cell death in A549 and a cervical cancer cell line HeLa while being non-toxic to normal MRC-5 lung cells. Higher expression of TfR in a number of cancer cells makes this Cyt c-Tf conjugate a promising candidate for inducing apoptosis in other TfR expressing cancer types.

## Materials and methods

Sulfosuccinimidyl 6-(3'-(2-pyridyldithio)propionamido)hexanoate (Sulfo-LC-SPDP) and Sulfosuccinimidyl-4-(*N*-maleimidomethyl)cyclohexane-1-carboxylate (Sulfo-SMCC) were purchased from Thermo Scientific (Rockford, IL, USA). A549, HeLa, K562, and MRC-5 cells were obtained from ATCC (Manassas, VA, USA). Human serum Apo-Tf, horse Cyt c, and other buffers and salts were obtained from Sigma-Aldrich (St. Louis, MO, USA). Apo-Tf was further processed to obtain the iron bound form of Tf and the amount of bound iron was determined by the ferrozine assay as described [27]. Apo-Tf concentration was calculated by using the molar absorption coefficient of 93 × 10^3^ M^-1^ cm^-1^ at 278 nm [28]. The molar ratio of 2:1 for bound iron to Tf was used to confirm that both Tf iron binding sites were occupied.

### Preparation of the Cyt c-Tf conjugate

**Fig 1:**
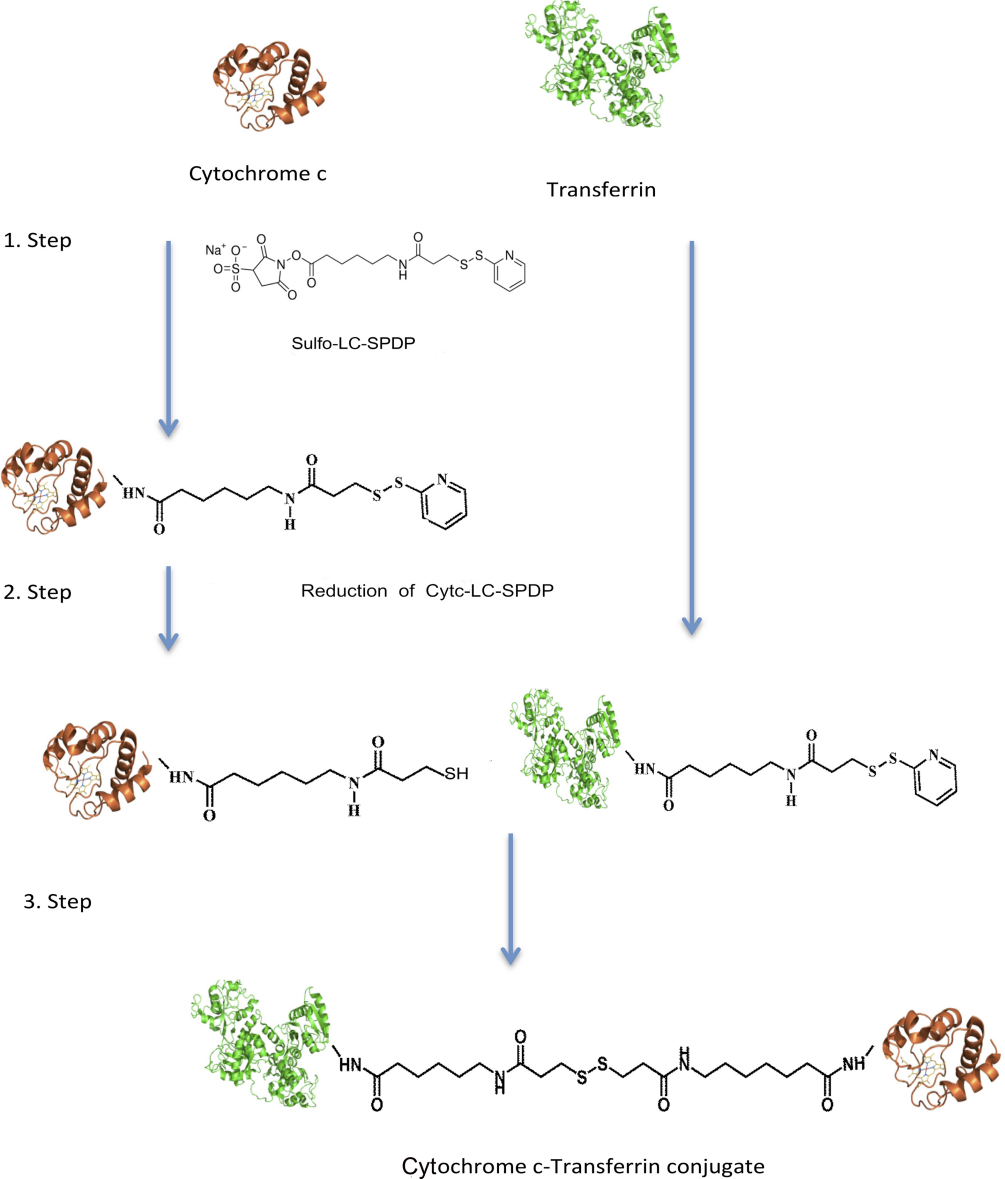
Three step synthesis of the Cyt c-Tf conjugate. In the first step, the proteins (molar ratio of 6 Cyt c:Tf) were reacted with the crosslinker (Sulfo-LC SPDP). In the second step, the LC-SPDP bound Cyt c was reduced to expose the–SH group. In the last step reduced Cyt c-LC-SPDP and Tf-LC-SPDP were incubated to obtain the final conjugate. The final protein conjugate was purified on a Superdex-200 10/300 column. The final conjugate contained a population of Cyt c-Tf conjugates with molar ratios of Cyt c to Tf varying from 1 to 5 Cyt c molecules attached to one Tf molecule.

**Fig 2:**
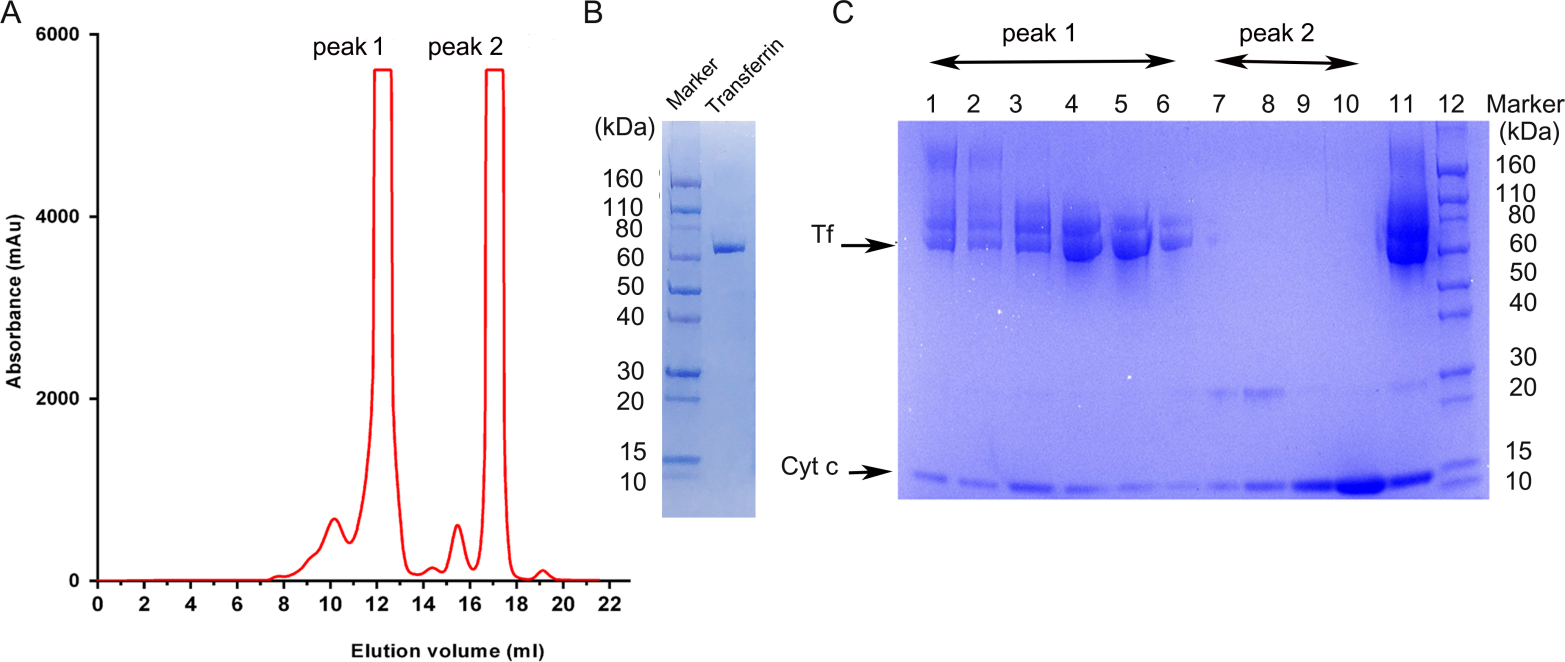
Purification of the Cyt c-Tf conjugate on a Superdex 200 increase gel filtration column. (A) Elution chromatogram from the Sephadex 200 column, peak 1 indicates the peak containing the conjugate. (B) Coomassie stained SDS PAGE 4–20% gradient gel loaded with unmodified transferrin and a protein molecular weight marker (C) Coomassie stained SDS PAGE 4–20% gradient gel of the fractions from the purification A; lanes 1–6 from the peak 1 in the chromatogram contain the conjugate (transferrin and cytochrome c co-eluting); lanes 7–10 corresponding to peak 2 in the chromatogram contain unconjugated free Cyt c. Lane 11 contains the concentrated conjugate before purification and lane 12 molecular weight markers.

A starting amount of 16 mg Tf and Cyt c at a 1:1 ratio (w:w) was used to synthesize the SPDP linked Cyt c-Tf conjugate by a three step process (**Fig 1**). In step one, 0.5 mM sulfo LC-SPDP was used to attach the SPDP conjugate to Tf and Cyt c in conjugation buffer (100 mM sodium phosphate, 150 mM NaCl, pH 7.5). In the second step, the SPDP attached to Cyt c was reduced with 5 mM tris(2-carboxyethyl)phosphine (TCEP) for 10 min at 37°C. After each step, the product was purified by passing through a desalting column pre-equilibrated with the conjugation buffer to remove excess of unreacted reagents. In the third step, the products of step two, i.e., the SPDP attached and subsequently reduced Cyt c and the SPDP attached Tf were mixed and incubated overnight at room temperature. The Cyt c-Tf neoconjugate was purified on a Superdex 200 increase 10/300 gel filtration column (**Fig 2A**). The final purified neoconjugate after the Superdex 200 column was analyzed on a SDS PAGE gradient gel 4–20% (**Fig 2B**).

A non reducible form of the Cyt c-Tf conjugate was synthesize using the SMCC crosslinker following the instructions of the manufacturer. Briefly, 8 mg of Cyt c was incubated in PBS buffer with a 20 molar excess of the crosslinker for one hour at RT. Excess of crosslinker was removed by passing the reaction mixture through a Hi-Trap column. The resulting SMCC linked Cyt c was incubated with a total of 8 mg of Tf in PBS buffer at RT over night. Finally, the conjugate was purified on a Superdex 200 column.

### Characterization of the Cyt c-Tf conjugate

#### MALDI TOF MS

The progress of the conjugation reaction, the composition and the stoichiometry of the final Cyt c-Tf conjugate was monitored by MALDI TOF MS. Products were desalted using a Hitrap desalting column prior to spotting on the MALDI plate. Mass spectrometric analysis was performed using the ABSCIEX 4800 Plus MALDI TOF/TOF™ Analyzer. To spot the samples a mixture of 1 μl of sample and 1μl of 5 mg/ml sinapic acid dissolved in a 50% (v/v) mixture of acetonitrile and MS grade water was used.

#### Gel filtration

The Cyt c-Tf conjugate was purified using a Superdex-200 increase (10/300) gel filtration column on an AKTA explorer (GE Health care) FPLC. The percentage of total protein that was converted to Cyt c-Tf conjugate was calculated based on the area under the peaks eluted from the Cyt c-Tf conjugate purification. Identity of the peaks were assigned based on the SDS-PAGE gel and MS of the corresponding fractions (**Fig 2 and S1 Table**).

#### Uv-Vis Spectroscopy

The stoichiometry of the Tf and Cyt c in the Cyt c-Tf conjugate was estimated based on the amount of protein calculated using molar extinction coefficient of 1.06 × 10^5^ M^−1^ cm^−1^ for the Cyt c at 410 nm and 1.04 × 10^5^ M^−1^ cm^−1^ for transferrin at 280 nm [26,27]. To check the 280 nm absorption due to Cyt c in the Cyt c-Tf conjugate, an equivalent amount of Cyt c was taken (based on equivalent 410 nm absorption) and its absorption was recorded on a NanoDrop 2000/2000c spectrophotometer (Thermo Scientific, CA, USA). As Cyt c contains only one tryptophan and therefore the absorption at 280 nm from this protein is negligible at 280 nm. The residual amount of 280 nm absorption from Cyt c in the conjugate due to other aromatic amino acids was subtracted from the total 280 nm absorption of the conjugate to calculate the amount of Tf in the conjugate. Similarly the 410 nm absorption from the Tf is negligible because it does not have a chromophore and the contribution of the 465 nm ligand metal charge transfer absorption is very low hence it was used to calculate the Cyt c [28].

#### Dynamic light scattering (DLS)

The hydrodynamic radius of Tf before and after the conjugation was determined by DLS using a DynaProTitan instrument (Wyatt Technology, CA, USA). All solutions were made in PBS at pH 7.4. The samples were centrifuged at 15,000 g for 10 min prior to the measurements. Data was analyzed using the Dynamic 6.7.6 software. Reduction of the conjugate was done separately using two reducing agents (TCEP and with glutathione) with a final concentration of 10 mM for varying time periods at 37°C. The change in the hydrodynamic radius of the conjugate following the reduction of the bond between Cyt c and Tf was followed via DLS measurement.

#### Circular dichroism (CD) spectroscopy

CD spectra were recorded using a Jasco high performance J-15000 CD spectrometer (Tokyo, Japan) at 25°C in 20 mM PBS at pH 7.4. The secondary structure (200 to 260 nm) and the tertiary structure (260 to 350 nm) of Cyt c was probed using a quartz cuvette of 1 mm path length at a concentration of 0.3 mg/ml and 2 mg/ml respectively. Similarly, Tf secondary and tertiary structure was probed at a concentration of 0.8 mg/ml and 3 mg/ml. Data was collected at a DIT of 4 seconds, a scan speed of 50 nm per minute, a bandwidth of 1 nm, and a data pitch of 0.1 nm. Spectra of buffer blanks were measured prior to the samples and subtracted digitally using the Spectral Manager (Jasco, Tokyo, Japan) software.

#### Immunofluorescence staining and confocal imaging

Internalization of the conjugate by A549 cancer cells was followed using immunofluorescence staining. Mitochondria in the A549 cells were localized using MitoTracker red dye (Thermo Scientific, Rockford, IL, USA), while Cyt c distribution was visualized using a FITC conjugated monoclonal antibody (A18512, Invitrogen, Frederick, MD, USA) as per the manufacturer instructions. Cells were washed with PBS and incubated with MitoTracker dye at a final concentration of 200 nM for 30 min at RT. Excess of the dye was removed by washing twice with PBS. After the staining of the mitochondria, Cyt c was stained using a FITC-conjugated Cyt c antibody (1:300 dilution, in 1% BSA) in both, control untreated cells and cells treated with the Cyt c-TF conjugate. Cells were fixed with 3.7% formaldehyde for 15 min and later washed twice with PBS. Fixed cells were permeabilized with 0.1% Triton X-100 dissolved in PBS and incubated with 1% BSA for 1 h at room temperature to block nonspecific binding prior to incubation with the Cyt c antibody. Cells were evenly covered with glycerol and stored in the dark until the confocal imaging was done.

#### Endosomal localization

To test if the internalized conjugate of Cyt c was localized in the endosome 2 x 10^4^ A549 cells were plated per well on a four well microscopic side. Cells were treated with the 50 μM of the Cyt c-Tf conjugate for 12 h. After the treatment, cells were fixed, permeabilized and blocked as described in the section for internalization studies. For localization of early endosomes a 1: 200 dilution of the EEA1 antibody (PA1063A) from ThermoFisher Scientific, was used. To localize the Cyt c-Tf conjugate a 1:300 (in 1% BSA) dilution of the FITC conjugated primary antibody against Cyt c was used. After incubation of the blocked cells with the mixture of the primary antibodies of EEA1 and Cyt c, cells were washed three times with PBST and incubated with a 1:2000 dilution of the Alexa Fluor-555 secondary antibody (A27039, Thermo Scientific, Rockford, IL, USA) for 1 h at room temperature. After incubation with the secondary antibody, cells were washed with PBST three times and finally the cells were incubated with DAPI for 2 minutes at room temperature to stain the nuclei. After nuclear staining, the cells were washed and evenly covered with glycerol and stored at 4°C in the dark until confocal imaging was done.

#### Cytotoxicity assay

A cell viability assay (CellTiter 96 aqueous one solution) from Promega (Madison, WI, USA) was used to measure the IC_50_ value for the Cyt c-Tf in the A549 cancer cells. In 200 μl media containing 10% FBS, DMEM supplemented with 1% penicillin and streptomycin, 5 x 10^3^ cells were plated in each well using a 96 well plate for attachment. After overnight growth, cells were incubated with different concentrations of the conjugate for a period of 24 h at 37°C. At the end of incubation 20 μl of MTS reagent was added and incubated for 1 h at 37°C. The absorption was recorded at 495 nm using a multiwall plate reader Tecan M200 (Tecan Deutschland GmbH, Crailsheim, Germany). Wells containing media alone were used as blanks to subtract absorption by media components. The absorption from wells containing cells without conjugate was considered 100% viable and all readings were normalized against it. Once the IC_50_ was determined, additional cell toxicity assays were performed in the lung cell line MRC5, HeLa cells and the chronic myeloid leukemia cell line K562 using the same procedure as described above for A549 cells.

#### Caspase-3/7 activity assay

A549 cells were grown to 80% confluency and harvested as explained above. The cells were then seeded (1 × 10^5^ cells/ml) into black 96-well plates (clear bottom) with 100 μl of DMEM per well. After 24 h, cells were incubated with a final concentration of 17.7 μM of the conjugate samples prepared in PBS for 24 h. Subsequently caspase-3 activity was measured using the *Apo-ONE*^*®*^ *Homogeneous Caspase-3/7 Assay* (Promega, Madison, WI, USA). After completion of the incubation, the media was removed and 50 μl of fresh DMEM (without phenol red to diminish background interference) and 50 μl of Apo-ONE^®^ Caspase-3/7 Reagent (1:100 substrate diluted in lysis buffer) was added to each well. The plate was gently mixed using a plate shaker at 150 rpm for 1 h at room temperature. After 1 h, the fluorescence of each well was measured (excitation at 485 nm, emission at 527 nm) in the Tecan M200 plate reader.

#### Caspase-9 activity assay

To perform the caspase-9 activation assay in A549 cells, a total of 10^4^ cells were seeded in a 100 μl volume of DMEM media using a black clear bottom 96 well plate. After 24 h, cells were incubated with a final concentration of 50 μM of the conjugate prepared in PBS for 24 h. Subsequently caspase-9 activity was measured by the luminescence measurement on a Tecan M200 plate reader after incubation at RT for 1 h and adding 100 μl of the freshly reconstituted Caspase-9 reagent from the Caspase-Glo^®^ 9 kit (Promega, Madison, WI, USA).

## Results

### Preparation and characterization of the Cyt c-Tf conjugate

The Cyt c-Tf conjugate was synthesized as shown in **Fig 1**. Products of the conjugation crosslinking steps 1 and 2 were analyzed by MALDI TOF MS. The MS data confirmed that during step 1 and 2 Cyt c and the Tf were successfully modified. Both, Cyt c and Tf showed an increment in their sizes by ~202 Da or its multiple as expected (**S1 Fig and S2 Fig**). The final conjugation product was purified on a gel filtration column (**Fig 2A**). The fractions from the Superdex-200 gel filtration column were analyzed on a denaturing 4–20% gradient polyacrylamide gel to localize fractions containing the Cyt c-Tf conjugate. Co-elution of Cyt c and the Tf from the gel filtration column in one of the peak fractions visualized by stained gel indicated that they were successfully linked (**Fig 2**). In the fractions containing the conjugate, one slowly migrating band, corresponding to Tf, and a faster migrating band, corresponding to Cyt c, were observed in the Coomassie stained gel (**Fig 2C**). In comparison to Cyt c and Tf, Cyt c-Tf elutes earlier from the size exclusion gel chromatography column as expected. Based on the elution profile analysis and SDS-PAGE gel 49% of the final purified product contains the Cyt c-Tf conjugate (**Fig 2 and S1 Table**).

**Fig 3:**
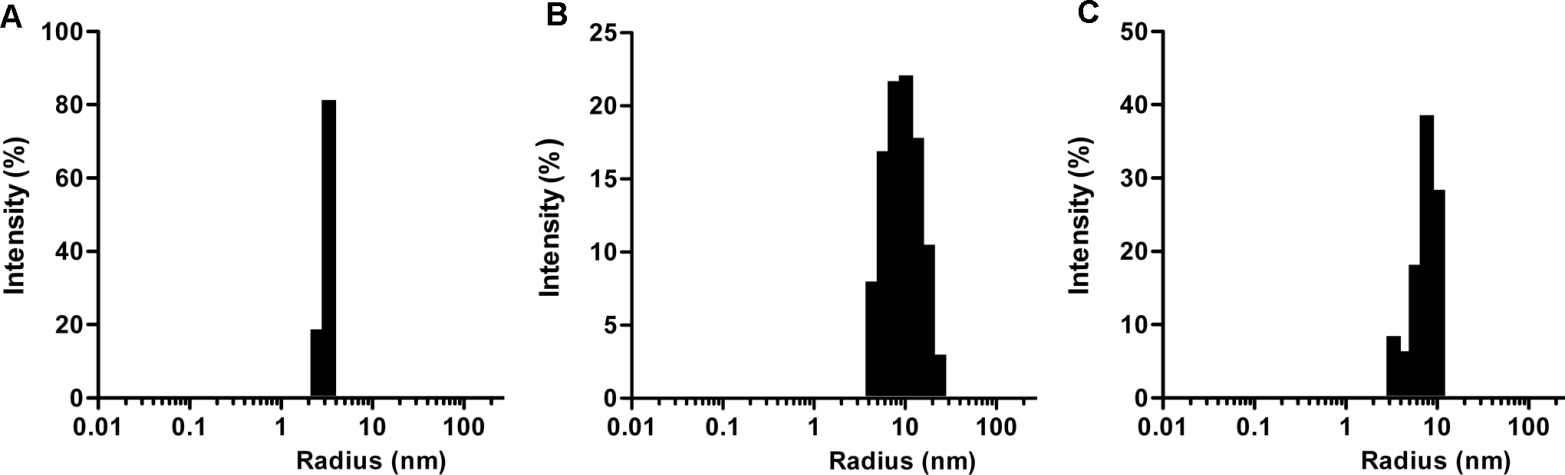
Size determination (hydrodynamic radii) using dynamic light scattering measurements. (A) Tf (B) Cyt c-Tf conjugate (C) Reduced Cyt c-Tf conjugate after incubation with 10 mM of TCEP.

Because the size of a drug conjugate can greatly influence its pharmacokinetics and for general characterization purposes, we determined its size using dynamic light scattering. The hydrodynamic radius of the conjugate was estimated to be ~10 nm, a size that would avoid its ready filtration by kidney but still smaller than 200 nm, a size that would promote clearance by immune cells (**Fig 3**) [29]. Our measured hydrodynamic radius of Tf was 3.9 ± 0.15 nm similar to reported values, validating our technique [30]. The molar ratio of Cyt c:Tf calculated based on UV-vis absorption was ~2.3. This number represents the average molar ratio of the Cyt c to Tf molecules in the conjugate. The MALDI-MS data of the conjugate further reveals the presence of different conjugate species containing from a minimum of one and up to a maximum of five Cyt c per Tf molecule (**S3 Fig**). The impact of crosslinking on the structural stability of the Cyt c and Tf was studied using CD spectroscopy. The comparison of CD spectra of unmodified Cyt c and Tf with their cross-linked forms revealed that the conditions used for crosslinking did not perturb the secondary or tertiary structure of the crosslinked proteins (**S4 Fig**).

The cytosol is reducing in nature and consequently the development of systems with a redox sensitive switch has been a major area of focus in the development of smart drug released systems[31]. To check the reducibility and release of Cyt c under intracellular reducing conditions, the Cyt c-Tf conjugate was incubated with 10 mM glutathione [32]. The appearance of two peaks in the DLS measurement after incubation under two different reducing conditions (TCEP and glutathione) confirmed the reduction of the disulfide bond in the linker and release of Cyt c (**Fig 3 and S5 Fig**). However, in case of the non-reducible form of conjugate hydrodynamic radius of conjugate remains unchanged after incubation with 10 mM TECEP as expected (**S6 Fig**). Additionally, the reduction of the conjugate was further validated by purification of the reduced conjugate on a Superdex 200 gel filtration column. The FPLC elution profile from the purification resolved the conjugate peak into two individual constituent protein peaks, therefore, confirming the successful breakage of disulfide bond on reduction (**S7 Fig**).

### Uptake of the Cyt c-Tf conjugate by A549 cells

**Fig 4:**
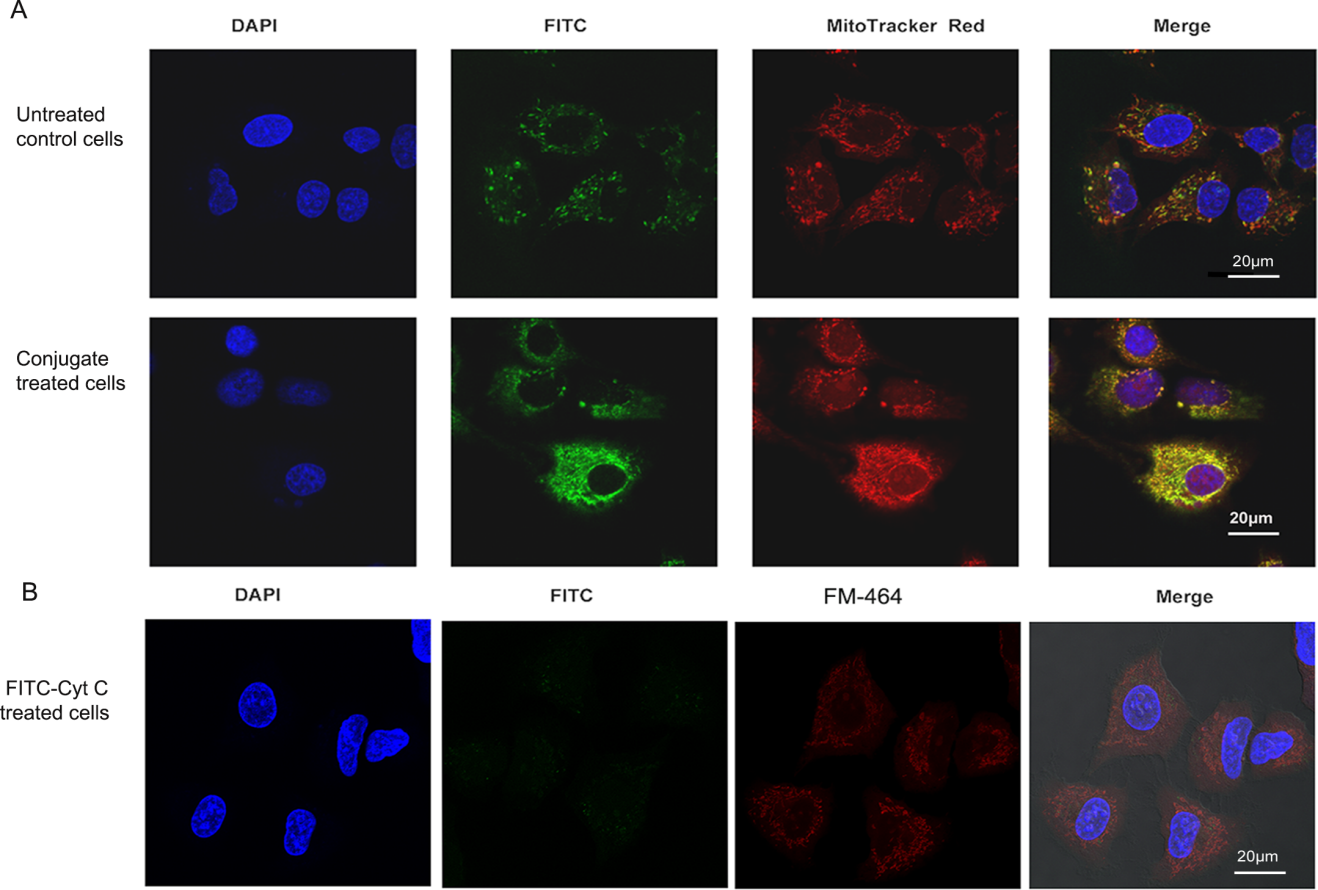
Confocal images showing the internalization of the Cyt c-Tf conjugate by A549 cancer cells. (A) Nuclei were stained with DAPI; Cyt c was stained with a FITC labeled anti-Cyt c antibody; mitochondria were stained with MitoTracker Red. In FITC panels, control cells (top panel) show that Cyt c is localized (green punctate) while in the treated cells (lower panel) increased green signal indicates successful delivery of Cyt c to the cytoplasm. (B) A549 cells treated with FITC-Cyt c and stained with DAPI and membrane staining FM-464.

**Fig 5:**
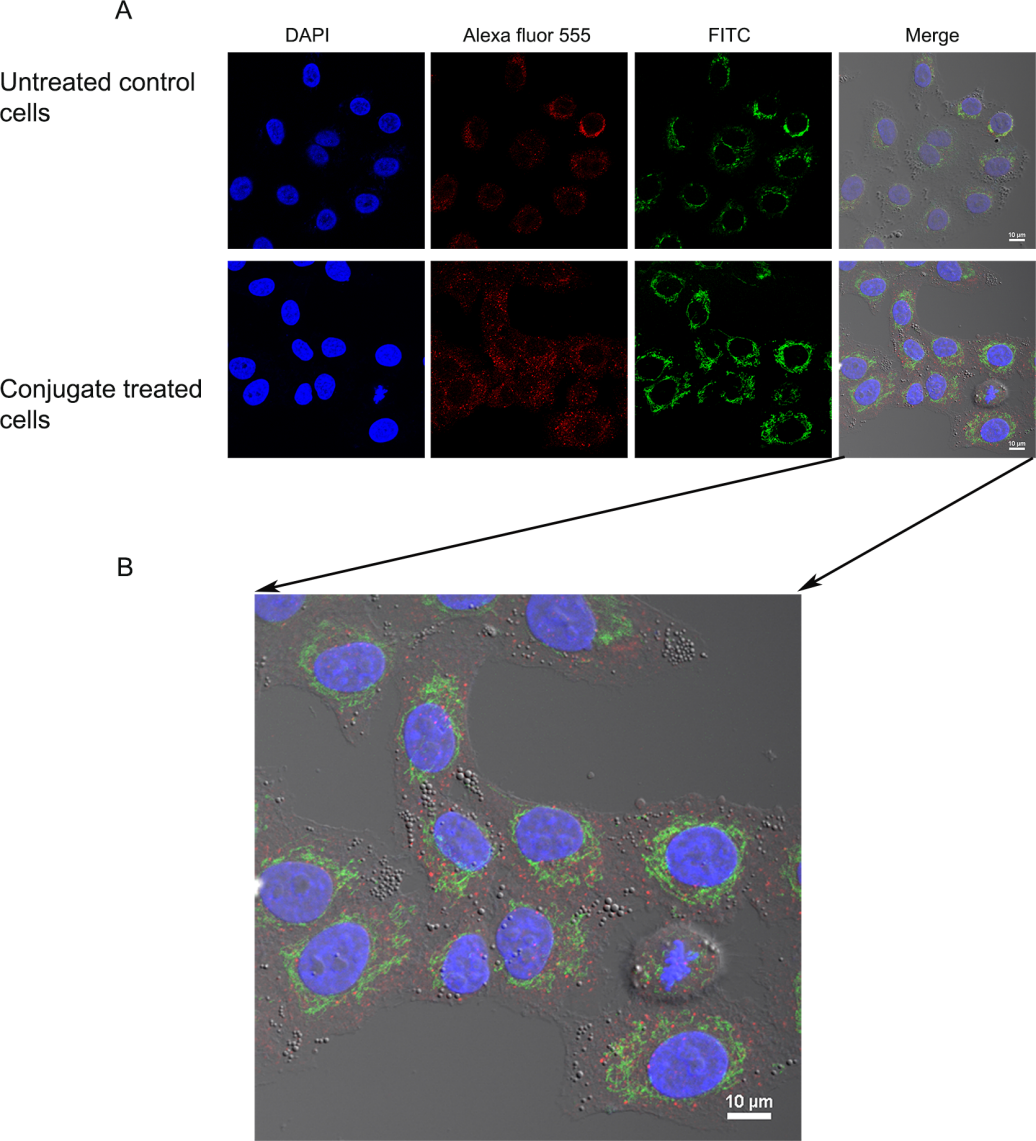
Endosomal localization of Cytc-Tf conjugate in A549 cells using confocal imaging. (A) A549 cells were treated with the Cyt c-Tf conjugate for 12 h and endosome localization was probed with an endosomal marker antibody (primary antibody against EEA1 and a secondary antibody conjugated with Alexafluor-555) and Cyt c was localized with an anti-Cyt c FITC labeled antibody. The upper panel shows untreated cells and the lower panel the cells treated with Cyt c-Tf. (B) Merged image of A549 cells treated with conjugate enlarged to show nonover lapping green (Cyt c) and red (endosomes) signal.

Many cancer cells show enhanced expression of Tfr and consequently Tfr mediated endocytosis has been used to deliver different drugs by conjugating them to Tf [33]. We validated Tf uptake by A549 cells by testing the internalization of FITC labeled Tf. Fluorescence images showed green punctate in the incubated cells indicating successful uptake of Tf (**S8 Fig**). After confirming that Tf uptake is functional in A549 cells we further studied the conjugate uptake by localizing the Cyt c using an FITC labeled Cyt c antibody and the mitochondria specific stain MitoTracker Red. The untreated A549 cells stained with FITC-conjugated Cyt c antibody showed green punctate fluorescence indicating the localized distribution of the endogenous Cyt c (**Fig 4**). When these cells were counterstained with MitoTracker Red, colocalization of red and green fluorescence validated that in untreated cells Cyt c was localized in the mitochondria and, as anticipated, no Cyt c was localized in the cytoplasm. In contrast, in cells treated with the Cyt c-Tf conjugate and stained with the FITC conjugated Cyt c antibody, green fluorescence was found throughout the cytoplasm (**Fig 4**). This result shows that in the conjugate treated cells Cyt c was successfully delivered to the cytoplasm. Compared to untreated cells the conjugate treated cells show a higher intensity of Cyt c staining (**Fig 4 and Fig 5**) and better colocalization with the mitochondrial signal indicating cellular uptake of conjugated Cyt c (**Fig 4B**). There are several in vitro studies showing that exogenous Cyt c can enter mitochondria and even partially rescue the compromised redox transition state of FAS pathway activated mitochondria [33–35]. Additionally, Martinou et al. have shown that the mitochondrial Cyt c loss during apoptosis in nerve growth factor (NGF) deprived sympathetic neurons is reversible upon addition of NFG to the neurons [36]. Based on these reports, the possibility that some of the exogenous Cyt c from the conjugate could have entered the conjugate treated cell’s mitochondria cannot be ruled out. In comparison to the Cyt c-Tf conjugate treated cells, the control cells treated with FITC-Cyt c do not show any FITC signal in the cytoplasm. This result indicates that Tf conjugation is required for Cyt c to enter the cytoplasm (**Fig 4B**).

To study the fate of the Cyt c-Tf conjugate its endosomal localization was followed using double immune staining with a FITC conjugated Cyt c antibody and an antibody as endosome marker, EEA1 (**Fig 5**). Our confocal data suggest that the cells treated with the conjugate internalized Cyt c and in the double staining of the cells with EEA1 and the Cyt c antibody we observed that the red and the green fluorescence signal did not overlap indicating that most of the delivered Cyt c could escape the endosome (**Fig 5B**).

### Cytotoxicity of Cyt c-Tf conjugate

**Fig 6:**
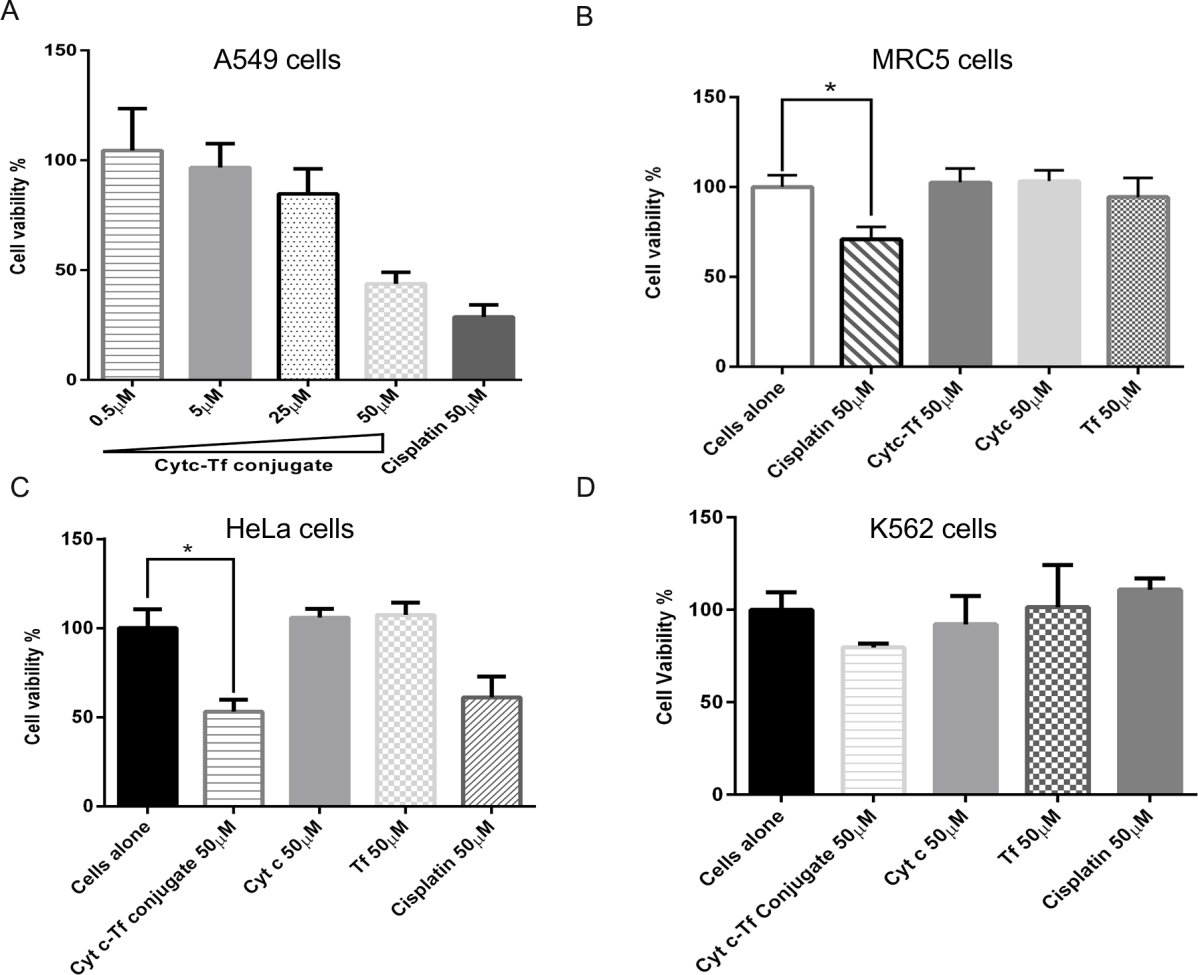
Cell viability assays in a lung cancer cell line (A549), in normal (MRC5) lung cells, a cervical cancer cells (HeLa) and in a chronic myleloid leukemia (K562) cells upon incubation with the Cyt c-Tf conjugate. (A) Cell viability assay (MTS) in A549 cells to determine the IC_50_ value of Cyt c-Tf conjugate. Cells were treated with an increasing amount of Cyt c-TF. (B) Equimolar concentrations (50 μM) of cisplatin, Cyt c-Tf, Cyt c, and Tf were incubated with MRC5 cells (C) HeLa and (D) K562 cells and subsequently cell viability was tested by MTS assay. * Indicates P<0.05, significant difference from control cells as assessed by two tailed unpaired t-test with n = 4.

Cytotoxicity of the Cyt c-Tf conjugate in A549 cells was tested using an MTS assay. This assay is based on the ability of viable cells to reduce the tetrazolium compound to a colored formazan dye that can be quantitated by measuring its absorption at 495 nm. The results show that the Cyt c-Tf conjugate IC_50_ value is similar to the reported IC_50_ value of cisplatin [37] (**Fig 6A**). To compare the toxicity of cisplatin and the Cytc-Tf conjugate in the normal lung cells, we tested the same dose (50 μM) of both in MRC-5 cells. Our results show that under similar conditions, compared to cisplatin, Cyt c-Tf was non-toxic to MRC-5 cells (**Fig 6B**). Tf conjugates have been successfully used in the past to deliver a different variety of drugs[21, 38]. The observed lack of toxicity in the normal cells could be explained by the differential expression of the TfR in normal cells compared to cancer cells [39]. To further validate the toxicity of the Cyt c-Tf conjugate in cell lines other than A549, the conjugate was tested in a human cervical cancer cell line, HeLa, and human chronic myeloid cell line, K562 (**Fig 6C and 6D**). In case of HeLa cells, a significant reduction in the cell viability was observed compared to the untreated cells upon incubation with 50 μM of the neoconjugate. However, as expected, in case of the K562 cells we did not see any statistical difference in the viability of the cells treated with the conjugate versus untreated cells. In K562 cells the mitochondrial apoptosis pathway is blocked due to a mutation as a result of a chromosomal translocation [40]. These results demonstrate that the developed targeted Cyt c-Tf conjugate could result in a powerful drug with a much better therapeutic index than drugs currently being used.

### Caspase-3 and Caspase-9 activation by the Cyt c-Tf conjugate in A549 cells

**Fig 7:**
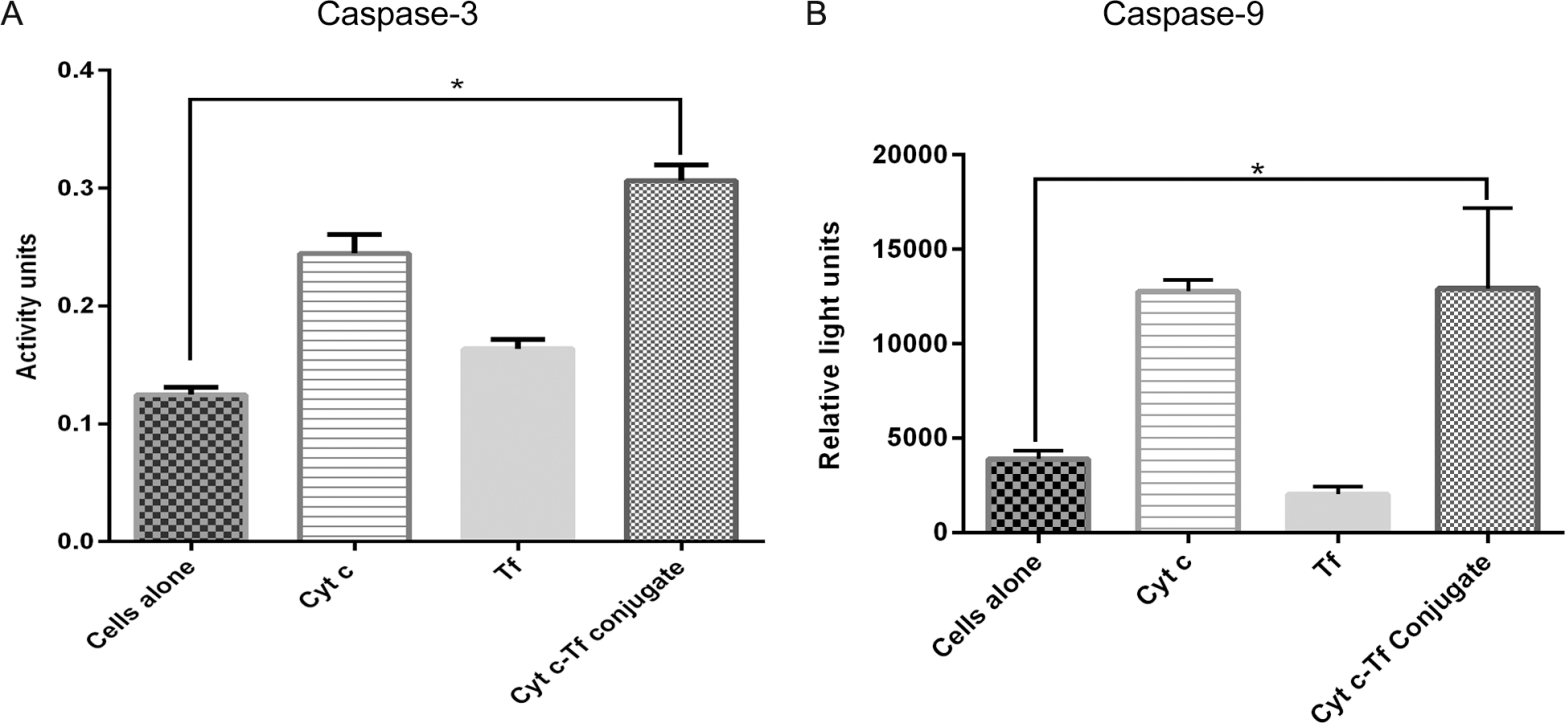
Activation of caspase-3 and caspase-9 using cell-free caspase assay. (A) Compared to the cells alone control, the Cyt c-Tf conjugate activates caspase-3 to a significantly greater extent indicating that the conjugate can induce apoptosis. (B) Cells treated with the Cyt c-Tf conjugate were able to activate the caspase-9 to a significantly greater extent compared to untreated cells or cells treated with Tf alone. * Indicates P<0.05, significant difference from control cells as assessed by two tailed unpaired t-test with n = 4.

In many types of cancer, the cells avoid cell death due to mutations along the apoptotic pathway. To test the potential of the Cyt c-Tf conjugate to induce apoptosis in vitro, an extracellular caspase-3 activation assay was performed. It was found that compared to the untreated cells, activation of caspase-3 was significantly higher in cells treated with the conjugate (**Fig 7**). This result indicates that during the conjugation the ability of the Cyt c to induce caspase-3 activation was not compromised. This result is significant as many of the surface lysines are known to be involved in the Apaf-1 Cyt c interaction, which is important for the activation of the caspase-3 [41, 42]. This result implies that conjugation does not necessarily adversely impact the Cyt c ability to induce apoptosis even though conjugation involves some of the lysines. It is possible that under the conditions used (low level of crosslinking) important Apaf-1 binding lysines remains available. In case of the mitochondrial apoptosis pathway caspase-9 and caspase-3 activation occurs in succession as part of the caspase activation cascade. Therefore, in addition to caspase-3, we tested the ability of the Cyt c-Tf conjugate to activate the caspase-9. Our results show that the conjugate was able to activate caspase-9 in A549 cells (**Fig 7B**) thus validating that the conjugate activates the mitochondrial apoptosis pathway.

## Discussion

Small molecules could be very effective in killing cancer cells but at the same time due to their off target effects and toxicities, they have a low therapeutic index thus damaging healthy cells at higher doses [43]. For example, although doxorubicin and vincristine are very potent drugs they too are limited in their dose due to their respective severe toxicities in cardio and neural cells [7, 44]. Nanoparticles provide various advantages, such as targeted delivery and increased drug loading. However, their use could result in additional challenges including, nanoparticle toxicity, clearance by the immune system, and unfavorable interaction with plasma proteins [45–47].

Due to excellent biocompatibility and selectivity, smaller protein conjugates are rapidly emerging as an alternative to synthetic nanoparticles or non-selective small drugs [43]. Herein we have synthesized and characterized a Cyt c-Tf conjugate and tested its uptake and apoptotic activity by an NSCLC cell line. We selected Cyt c due to its high stability, solubility and proven apoptosis inducing potential [8, 13]. However, cellular uptake of Cyt c is not known to occur except in advanced sepsis mice model where it is suggested to cross the leaky membrane of cardiomyocytes[35]. Therefore, in order to induce apoptosis in cancer cells in a targeted manner, a Tf based delivery system was developed. Additionally, due to its evolutionarily conserved nature, Cyt c could be less immunogenic whereas toxic proteins like ricin and other ribosome inhibiting proteins used in previously reported Tf-conjugates are highly immunogenic due to their plant origin [48]. In comparison to many nanoparticle-based delivery systems, the Cyt c-Tf conjugate does not involve the use of any external material as drug carrier thus potentially avoiding adverse immune responses. As in our conjugate protein drug is conjugated directly to serum human protein Tf we predict this conjugate to be well tolerated by the immune system.

For conjugation of Tf and Cyt c we selected Sulfo-LC SPDP as the cross-linker of choice over SPDP because SPDP is only soluble in DMSO or DMF-like organic solvents whereas Sulfo-LC SPDP is a water soluble cross-linker. Proteins retain their activity in their natural aqueous milieu whereas DMSO and other organic solvents could irreversibly inactivate proteins by denaturation. DMSO is known to strip the surface water of protein and compete for H-bonds resulting in protein denaturation [49]. Additionally, Sulfo-LC SPDP is a proven system for conjugating cargo to a wide variety of carriers under milder aqueous conditions to form a redox sensitive bond [10, 50, 51]. We confirmed this by our in vitro reduction experiment where we incubated the conjugate with the 10 mM glutathione and followed the subsequent breakage of the disulfide bond by dynamic light scattering. Additonally, incubation of the conjugate in acidic conditions similar to the endosome at pH 5.5 also resulted in breakage of the disulfide bond (**S5 Fig**). In comparison to glutathione or the acidic condition alone, a faster bond breakage of the conjugate with TCEP could be explained due to its high reduction power.

Our conjugate provides additional advantages as the majority of the Cyt c delivery methods developed so far which depend on folate-mediated targeting which excludes their use in A549 cells, a folate receptor negative cell line [52]. In this work, we have focused on the Tfr because this receptor is enriched in A549 cells and available to ligand based targeting strategies.

The drugs delivered via systems like Tf conjugates could be entrapped in the endosomes and this entrapment could render the drugs ineffective. The endosomal entrapment of protein drugs could result in their denaturation due to low pH and degradation by endosomal proteases like cathepsin D [53, 54]. Consequently an endosome release mechanism is often designed as part of the drug conjugates[55, 56]. Our confocal result suggests that Cyt c-Tf could escape endosomal entrapment (Fig 5). Although the exact mechanism of the endosomal escape of the Cyt c-Tf conjugate was not investigated, we hypothesize that this escape was facilitated by the high charge density of Cyt c. The high surface charge could have helped the protein escape the endosome in a manner proposed for cationic peptide escape from endosomes [55]. Highly charged cationic peptides and proteins are traditionally used to help endosomal escape [54]. In our system, this high charge density is built in as Cyt c has many positively charged residues on its surface. Furthermore, the low pH (as seen in endosome) is known to assist in Cyt c membrane interaction by enhancing the high surface charge in Cyt c by inducing protonation of lysine residues [55]. Peptides derived from Cyt c itself have been used as cell penetration peptides [57].

Additionally, Tf based conjugates are of specific interest due to the fact that Tf receptors are expressed on the endothelium of the blood vessels of the brain thus their conjugates have potential to cross the blood brain barrier (BBB) [58]. Wiley et al. have shown that Tf conjugated nanoparticles could be delivered across the BBB [59]. Interestingly, in a study done by Johnson et al. it was also shown that Cyt c could induce apoptosis specifically in glioblastoma and medulloblastoma cell lines whereas the primary neurons from the cerebellum and cortex were not affected [60]. Based on the Cyt c’s ability for preferential killing of glioblastoma cells and potential of Tf to deliver protein across the blood brain barrier, it would be an interesting proposition to test the effectiveness of this conjugate in relevant cell lines.

## Conclusions

Many of the drugs in use for the cancer treatment lack the specificity for targeting the cancer cells. To circumvent this issue, we presented here a conjugate of Cyt c and Tf and tested its delivery and toxicity in the A549 cells. Our in vitro cell study data suggest that the small globular apoptosis inducing protein Cyt c can be successfully delivered to the cytoplasm of target cells and activates the caspase cascade thus inducing cell death. We propose that for the future development of this conjugate design to allow for in vivo testing, surface modification with agents like PEG would be required to avoid collateral damage in normal cells like hepatocytes that express high levels of TfR[61, 62]. Our results suggest this conjugate would be effective in cancers with enhanced expression of TfR.

## Supporting information

S1 Fig MALDI MS spectra of Cyt c conjugated with Sulfo-LC SPDP. The difference in size of any two adjacent peaks is ~202 Da representing the size of the reduced Sulfo-LC SPDP part attached to Cyt c.
(TIF)

S2 Fig MALDI MS spectra of Tf conjugated with the Sulfo-LC SPDP. MS spectra of human serum Tf before (black) and after conjugation with Sulfo-LC SPDP (red) step 1 of **Fig 1**. The molecular weight of the portion of the Sulfo-LC SPDP crosslinker (unreduced form) attached to Tf is 311 Da as shown in the inset. The two peaks differ by a mass of 1940 Da indicating that ~6 molecules of the crosslinker are attached to the modified Tf.
(TIF)

S3 Fig MALDI-MS spectra of the purified Cyt C-Tf conjugate. MS spectra of the purified conjugate revealed five peaks corresponding to the mass of one Tf molecule conjugated with 1 to 5 molecules of Cyt c.The difference in size of any two adjacent peaks is ~12.8 kDa which is equal to the combined mass of a Cyt c protein molecule attached with portions of the LC-SPDP crosslinker.
(TIF)

S4 Fig CD spectra of the unmodified and the crosslinked (S-LC-SPDP modified) Cyt c and Tf. Unmodified Cyt c and crosslinked Cyt c in secondary (A) and tertiary region (B). Unmodified Tf and crosslinked Tf in secondary (C) and tertiary region (D). (E) and (F) CD signals normalized and expressed as %.
(TIF)

S5 Fig Cyt c-Tf conjugate dissociates when incubated under acidic or reducing condition. Dynamic light scattering of the Cyt c-Tf conjugate at the beginning of incubation (A) and after 12 h of incubation at room temperature in 100 mM sodium acetate buffer (pH 5.5) (B) or 10 mM glutathione (C) Hydrodynamic radius of unmodified Cyt c (1.7nm) shown for comparison (D)After 12 h of incubation the conjugate dissociates into two peaks as seen under both acidic as well as reducing conditions.
(TIF)

S6 Fig Cyt c-Tf conjugate none reducible form (control) with Sulfo-SMCC crosslinker. DLS data showing the hydrodynamic radii of the conjugate before (A) and after (B) reduction with 10 mM TCEP.
(TIF)

S7 Fig FPLC elution profiles of unreduced and reduced Cyt c-Tf conjugate from Superdex 200 increase column. (A) Elution peak of the unreduced Cyt c-Tf conjugate (B) Upon reduction with 10 mM TCEP, the Cyt C- Tf peak further resolves in to two peaks of constituents, Cyt C and Tf.
(TIF)

S8 Fig FITC labeled transferrin showing TfR in A549 cells and MRC5 cells. (A) Chromatogram showing the elution of FITC labeled protein from a Superdex 200 column. FITC absorbs at 495 nm while Tf absorbs at 280 nm. (B) Confocal image of FITC labeled transferrin internalized by A549 cells. The nucleus was stained with DAPI. (C) Z-stack image of A549 cells with FITC channel to differentiate the surface bound and internalized transferrin. Green punctate indicates the internalized FITC labeled transferrin. (D) Confocal image of MRC5 cells with FITC chanel after treatment with FITC labeled Tf. Compared to A549 a more diffused and weaker FITC signal indicates a lower level of TfR in MRC5 cells.
(TIF)

S1 Table Peak area and retention volumes of the Cyt c-Tf purification. (DOCX)
