# Supplementary figures and images for: Inducing cell death in vitro in cancer cells by targeted delivery of cytochrome c via a transferrin conjugate
Source: PLoS One. 2018 Apr 12;13(4):e0195542. doi: 10.1371/journal.pone.0195542 (PMC5896948; doi:10.1371/journal.pone.0195542)

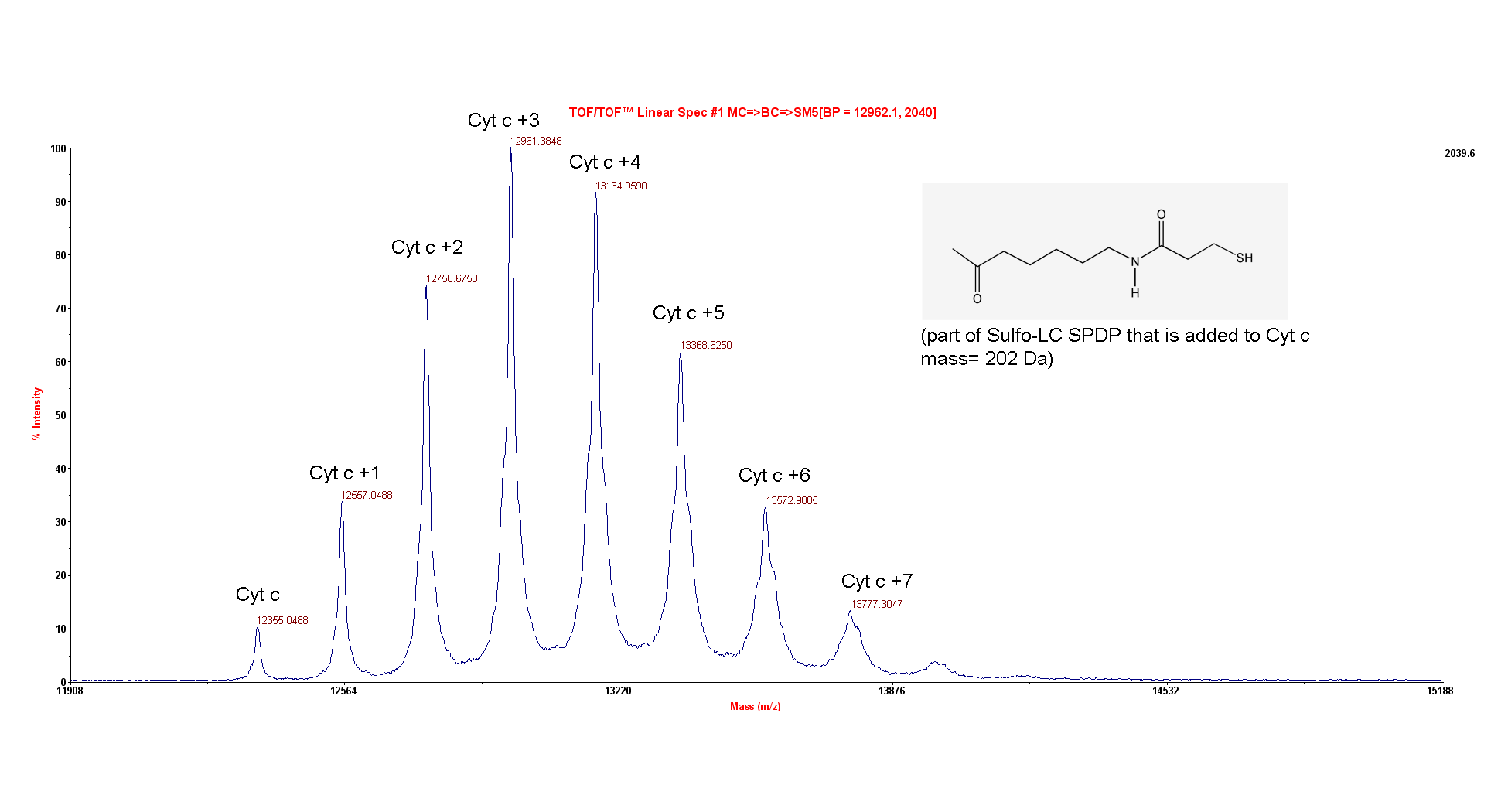

Supplement: S1 Fig — The difference in size of any two adjacent peaks is ~202 Da representing the size of the reduced Sulfo-LC SPDP part attached to Cyt c. (TIF) [file pone.0195542.s001.tif]

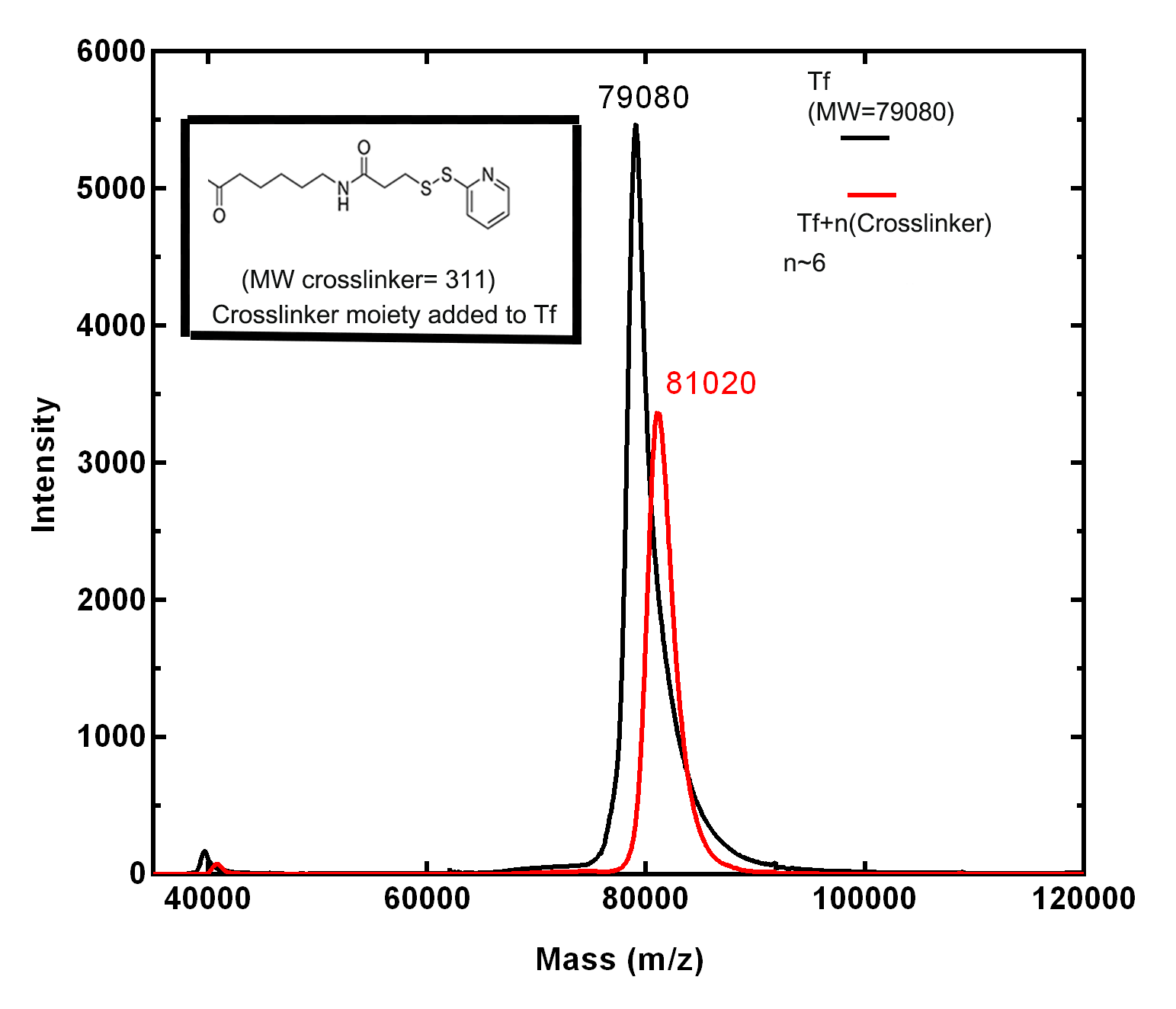

Supplement: S2 Fig — MS spectra of human serum Tf before (black) and after conjugation with Sulfo-LC SPDP (red) step 1 of Fig 1. The molecular weight of the portion of the Sulfo-LC SPDP crosslinker (unreduced form) attached to Tf is 311 Da as shown in the inset. The two peaks differ by a mass of 1940 Da indicating that ~6 molecules of the crosslinker are attached to the modified Tf. (TIF) [file pone.0195542.s002.tif]

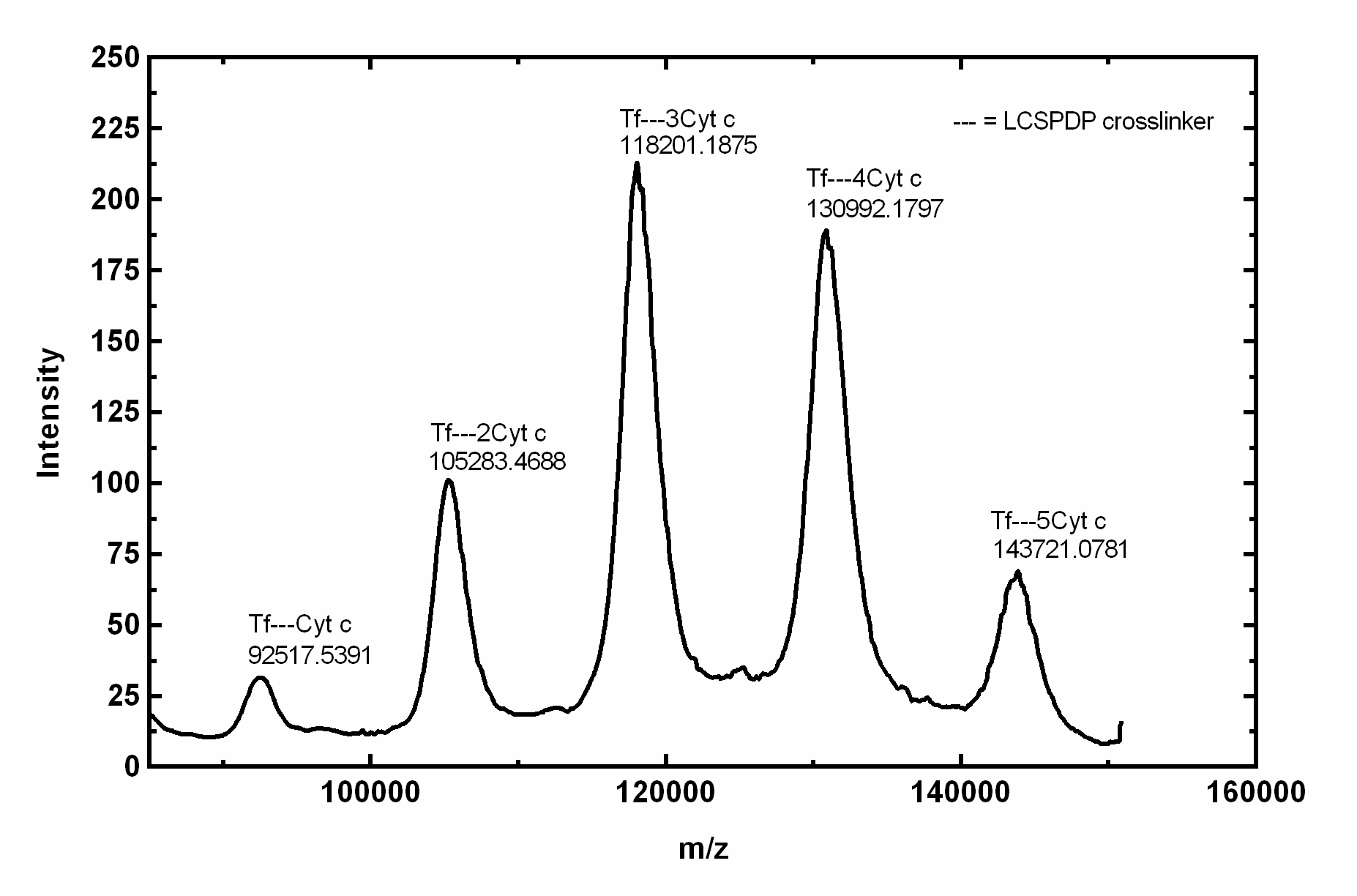

Supplement: S3 Fig — MS spectra of the purified conjugate revealed five peaks corresponding to the mass of one Tf molecule conjugated with 1 to 5 molecules of Cyt c.The difference in size of any two adjacent peaks is ~12.8 kDa which is equal to the combined mass of a Cyt c protein molecule attached with portions of the LC-SPDP crosslinker. (TIF) [file pone.0195542.s003.tif]

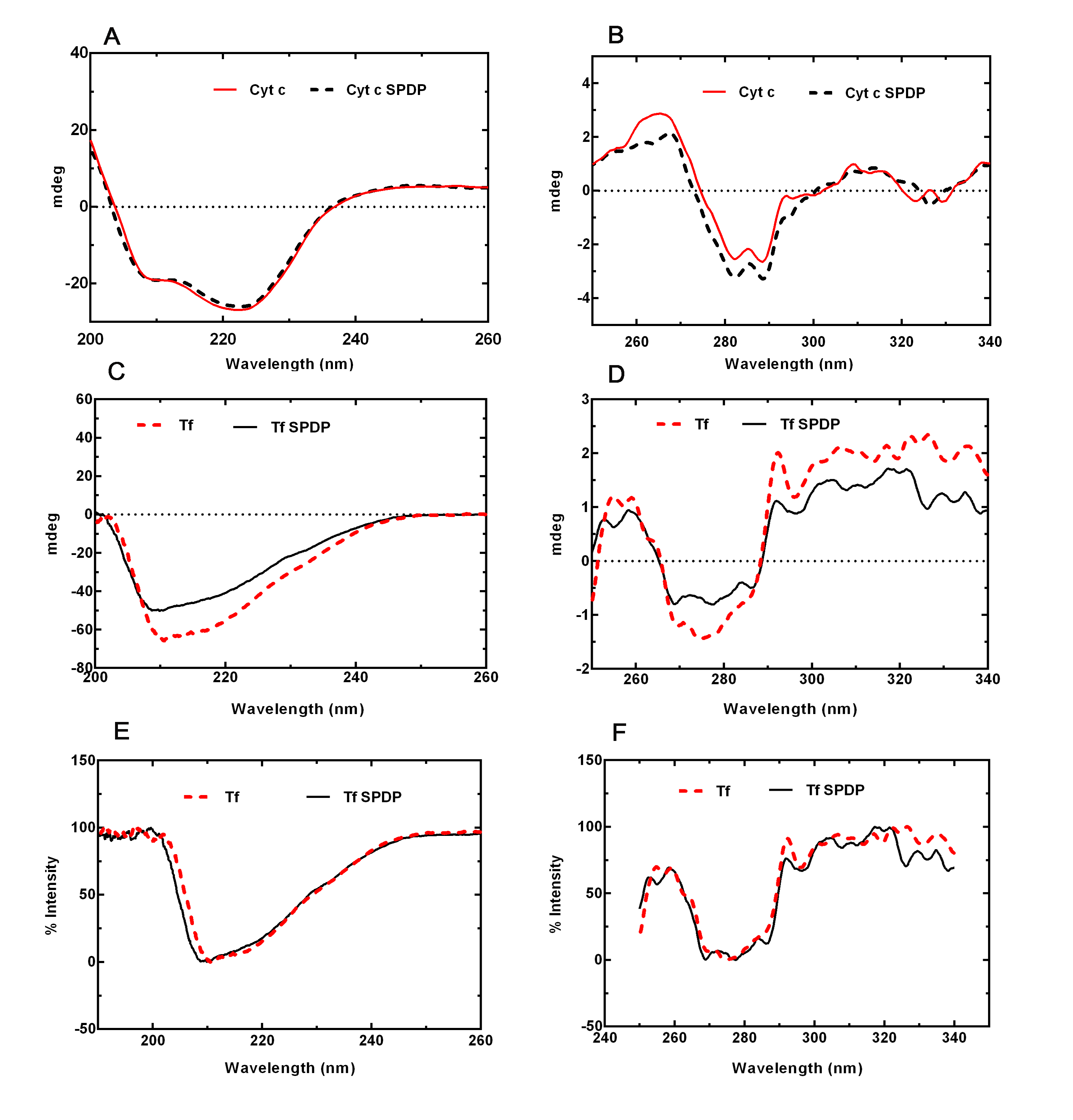

Supplement: S4 Fig — Unmodified Cyt c and crosslinked Cyt c in secondary (A) and tertiary region (B). Unmodified Tf and crosslinked Tf in secondary (C) and tertiary region (D). (E) and (F) CD signals normalized and expressed as %. (TIF) [file pone.0195542.s004.tif]

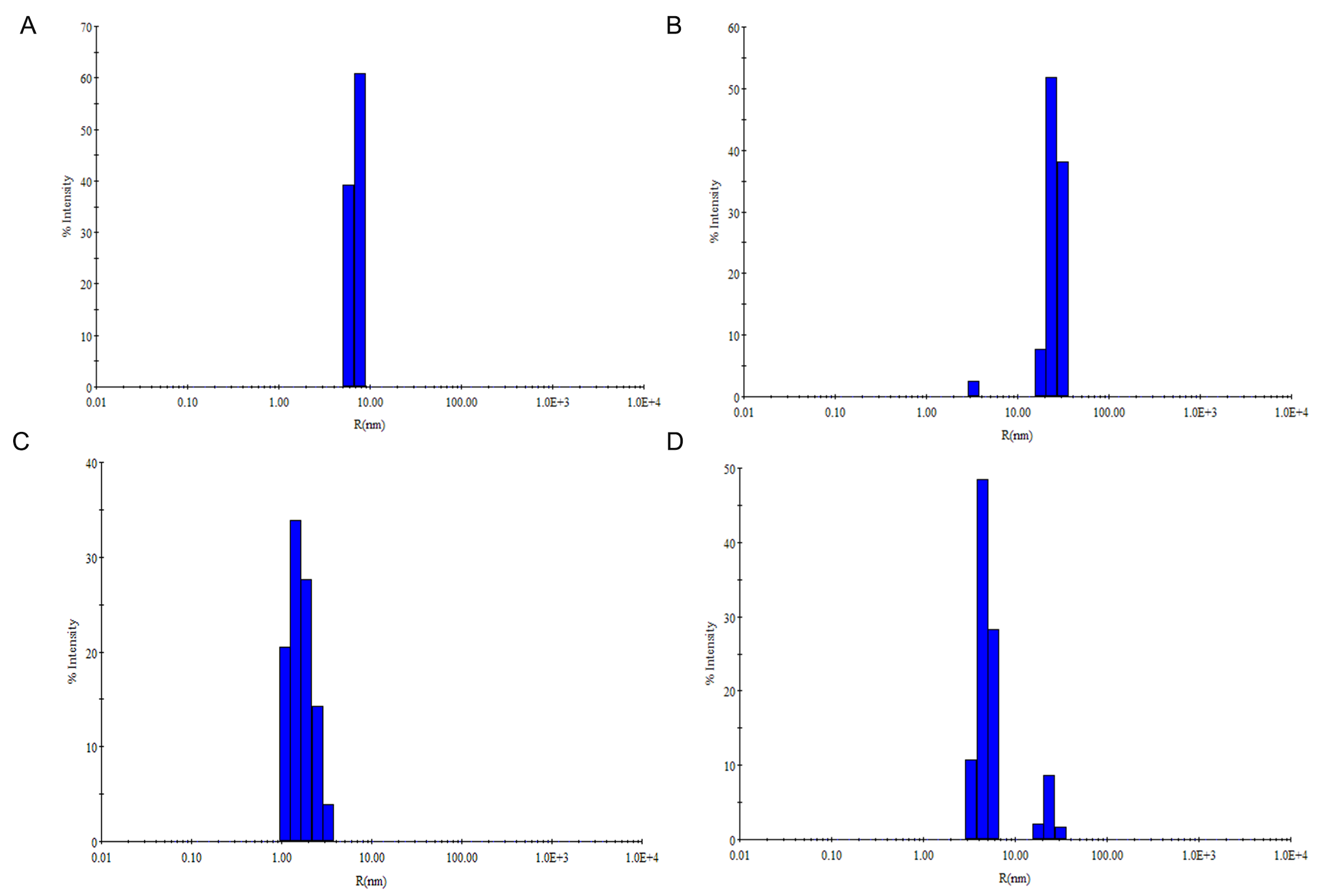

Supplement: S5 Fig — Dynamic light scattering of the Cyt c-Tf conjugate at the beginning of incubation (A) and after 12 h of incubation at room temperature in 100 mM sodium acetate buffer (pH 5.5) (B) or 10 mM glutathione (C) Hydrodynamic radius of unmodified Cyt c (1.7nm) shown for comparison (D)After 12 h of incubation the conjugate dissociates into two peaks as seen under both acidic as well as reducing conditions. (TIF) [file pone.0195542.s005.TIF]

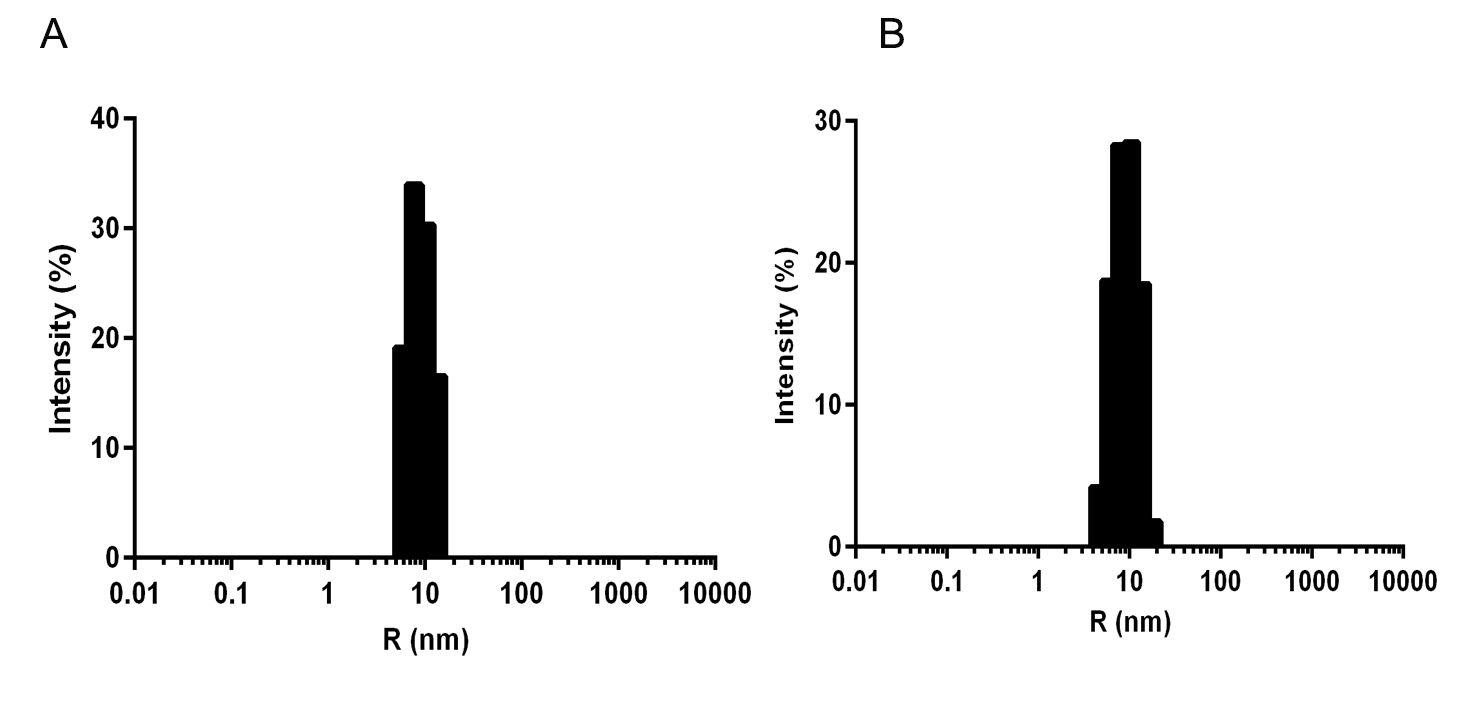

Supplement: S6 Fig — DLS data showing the hydrodynamic radii of the conjugate before (A) and after (B) reduction with 10 mM TCEP. (TIF) [file pone.0195542.s006.tif]

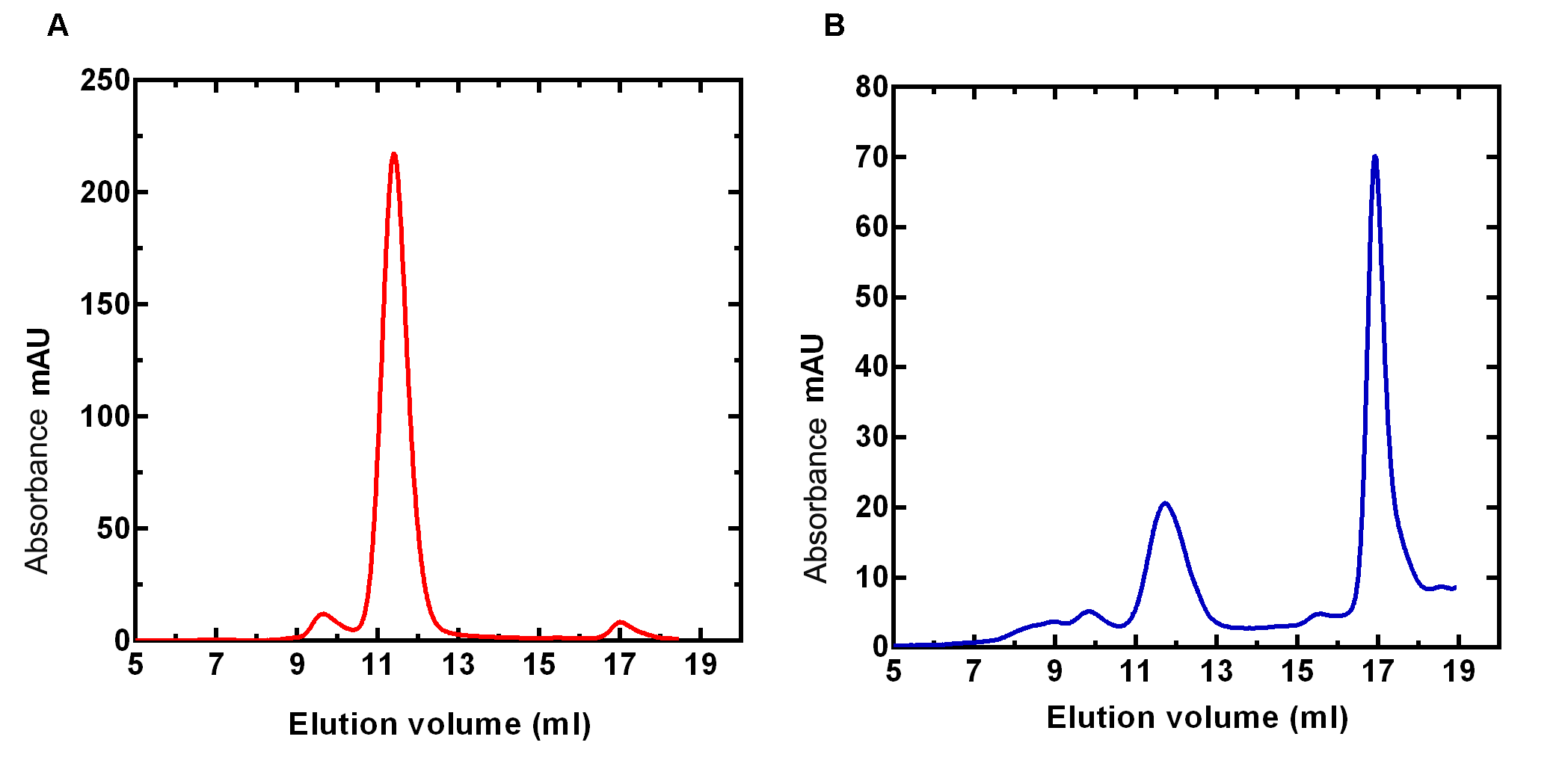

Supplement: S7 Fig — (A) Elution peak of the unreduced Cyt c-Tf conjugate (B) Upon reduction with 10 mM TCEP, the Cyt C- Tf peak further resolves in to two peaks of constituents, Cyt C and Tf. (TIF) [file pone.0195542.s007.tif]

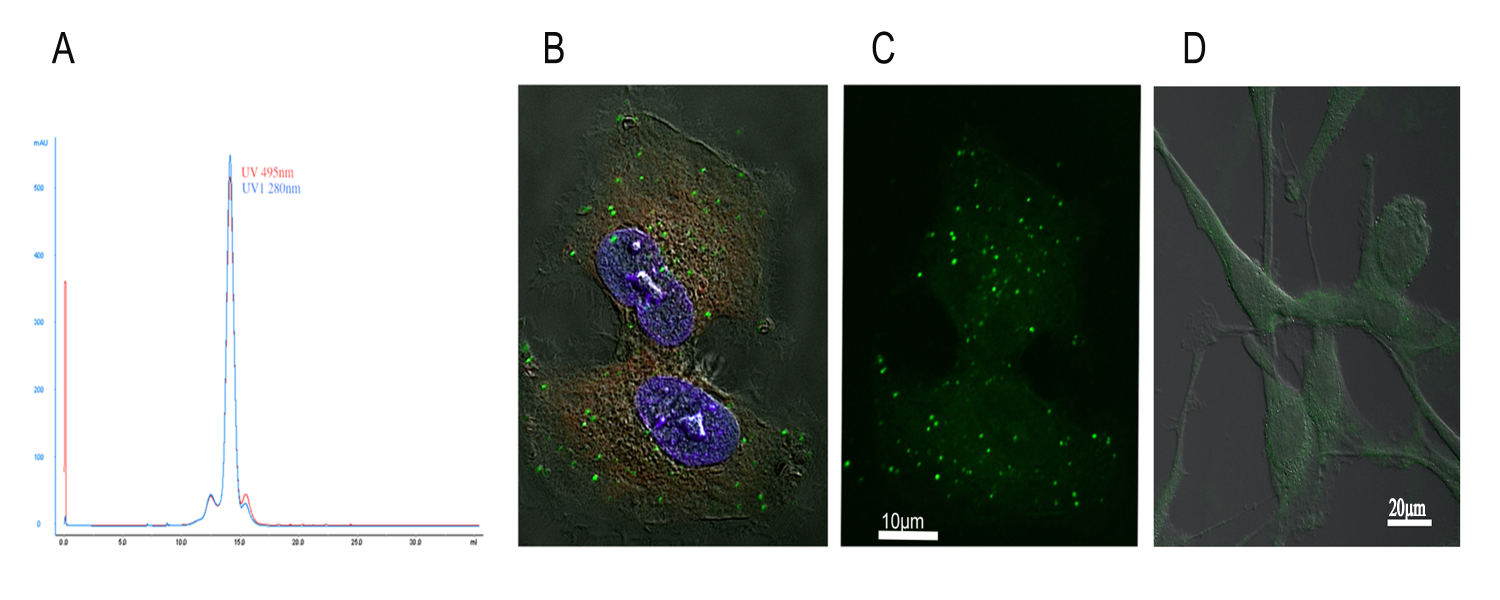

Supplement: S8 Fig — (A) Chromatogram showing the elution of FITC labeled protein from a Superdex 200 column. FITC absorbs at 495 nm while Tf absorbs at 280 nm. (B) Confocal image of FITC labeled transferrin internalized by A549 cells. The nucleus was stained with DAPI. (C) Z-stack image of A549 cells with FITC channel to differentiate the surface bound and internalized transferrin. Green punctate indicates the internalized FITC labeled transferrin. (D) Confocal image of MRC5 cells with FITC chanel after treatment with FITC labeled Tf. Compared to A549 a more diffused and weaker FITC signal indicates a lower level of TfR in MRC5 cells. (TIF) [file pone.0195542.s008.tif]
