# Supplementary material for: Inducing cell death in vitro in cancer cells by targeted delivery of cytochrome c via a transferrin conjugate
Source: PLoS One. 2018 Apr 12;13(4):e0195542. doi: 10.1371/journal.pone.0195542 (PMC5896948; doi:10.1371/journal.pone.0195542)
Supplement: S1 Table — (DOCX) [file pone.0195542.s009.docx]

S1 Table. Peak area and retention volumes of the Cyt c-Tf purification (Fig 1)

| **Peak name** | **Ret** | **Peak start** | **Peak end** | **Width** | **Peak Area** | **Peak area/Total area** |
| --- | --- | --- | --- | --- | --- | --- |
| Tf-Tf adducts | 10.16 | 8.66 | 10.82 | 2.16 | 597.23 | 5.25 |
| Cyt c-Tf conjugate | 12.62 | 10.82 | 13.29 | 2.47 | 5608.44 | 49.28 |
| Cyt c-Cyt c dimers | 15.46 | 14.88 | 16.13 | 1.25 | 322.41 | 2.83 |
| Cyt c | 17.43 | 16.13 | 18.07 | 1.94 | 4781.70 | 42.02 |

Ret = Retention volume (ml)
